# How Life Experience Shapes Cognitive Control Strategies: The Case of Air Traffic Control Training

**DOI:** 10.1371/journal.pone.0157731

**Published:** 2016-06-16

**Authors:** Sandra Arbula, Mariagrazia Capizzi, Nicoletta Lombardo, Antonino Vallesi

**Affiliations:** 1 Department of Neuroscience, Università degli Studi di Padova, Padova, Italy; 2 Human Factor Unit ENAV S.p.A., Forlì, Italy; University of Verona, ITALY

## Abstract

Although human flexible behavior relies on cognitive control, it would be implausible to assume that there is only one, general mode of cognitive control strategy adopted by all individuals. For instance, different reliance on proactive versus reactive control strategies could explain inter-individual variability. In particular, specific life experiences, like a highly demanding training for future Air Traffic Controllers (ATCs), could modulate cognitive control functions. A group of ATC trainees and a matched group of university students were tested longitudinally on task-switching and Stroop paradigms that allowed us to measure indices of cognitive control. The results showed that the ATCs, with respect to the control group, had substantially smaller mixing costs during long cue-target intervals (CTI) and a reduced Stroop interference effect. However, this advantage was present also prior to the training phase. Being more capable in managing multiple task sets and less distracted by interfering events suggests a more efficient selection and maintenance of task relevant information as an inherent characteristic of the ATC group, associated with proactive control. Critically, the training that the ATCs underwent improved their accuracy in general and reduced response time switching costs during short CTIs only. These results indicate a training-induced change in reactive control, which is described as a transient process in charge of stimulus-driven task detection and resolution. This experience-based enhancement of reactive control strategy denotes how cognitive control and executive functions in general can be shaped by real-life training and underlines the importance of experience in explaining inter-individual variability in cognitive functioning.

## Introduction

The uniqueness of human cognition derives from the flexibility in adapting thoughts and behaviors to rapidly changing internal and external states. Although there is a general agreement that this flexibility leans upon specialized cognitive control mechanisms that allow goal-directed behavior, it would be implausible to assume that all individuals rely equally on these mechanisms and adopt always the same strategies when acting in a goal-directed manner. To account for the empirically observed variability in cognitive control function, Braver [1] proposed the Dual Mechanism of Control (DMC) framework, which distinguishes two cognitive control modes: proactive and reactive. The former is characterized by sustained and anticipatory maintenance of goal-relevant information, while the latter is described as a transient, bottom-up reactivation of behavioral goals. According to the model, both inter- and intra-individual variability can be better understood by assuming different reliance on proactive and reactive control strategies. Despite a large interest in explaining the sources of individual variability, little is known on the development of different cognitive control processes and how they are shaped by specific life experiences.

The impact of different life experiences, like taxi driving [2], language learning [3–5], or music skills [6], has been addressed in studies investigating inter-individual variability in brain structure and function. However, all these studies cross-sectionally compare highly specialized individuals with a group of non-experts and this type of approach does not allow conclusions on the origins of this variability. Exploring how cognitive control functions are being modulated by experience in young adults requires a combination of a cross-sectional and a longitudinal approach in order to discriminate pre-existent abilities from acquired ones. The number of studies implementing a longitudinal approach to explore real-life training-related modulation of cognitive functions in young adults is slowly growing (e.g., simultaneous interpreters [7,8], meditation practitioners [9–11]). For this purpose, in the present study we will focus on Air Traffic Controllers (ATCs) whose profession is supposed to require extensive engagement of cognitive control and executive functions in general [12] but which, quite surprisingly, has not been the focus of much empirical research in cognitive psychology up to now. In order to investigate whether and how specific cognitive control processes get boosted during the ATC training, we tested ATC trainees both before and after their training period and compared their performance to that of a carefully matched control group.

Computational models have been put forward to describe the cognitive nature of the Air Traffic Control (ATC) profession and most of them include four major processes required in ATC: planning, implementation, monitoring of plan goals and evaluation [13]. However, within this profession there are different types of controllers responsible for different airspace divisions. Given that the first training phase is performed in airport tower simulators and on radar displays, we can hypothesize that, regardless of the specific cognitive process required, dealing with the high complexity of the environment is the first challenge they will encounter. As suggested by Koros and colleagues [14], in order to mitigate complexity ATCs have to learn different strategies and how to select them based on the traffic context, weather conditions, fatigue level, and other factors that determine the difficulty of the situation. A second major constraint they have to cope with is time: they are often provided with short decision-making time and have high demands for prompt actions.

Along these lines, we can assume that this type of training will improve behavioral flexibility by boosting advance task preparation, strategy switching and rapid task implementation. Moreover, because of the amount of information they have to keep track of, we could hypothesize improvements in short-term and working memory, and furthermore in resistance to interference from irrelevant information. Consequently, we measured ATCs’ performance, both before and after the training phase, on memory, task-switching and Stroop tasks. All these tasks tapped some of the cognitive control and memory abilities that could most likely be enhanced by the training. Specifically, the selection of these tasks was guided by models proposed in the ATC literature that outline the involvement of cognitive processes in this demanding profession [15]. Moreover, previous research has adopted a similar task battery as that used here in order to explore whether experienced ATCs show diminished age-related decline [16]. In the next section, we will describe in detail what cognitive measures were employed and how exactly did they relate to the training the ATCs underwent.

We used a cued task-switching paradigm that allowed us to assess different features of goal-directed behavior. In particular, by comparing performance on different types of trials (i.e., single-task, repeat and switch trials) we measured two different behavioral costs, namely mixing costs (i.e., performance difference between repeat and single-task trials) and switching costs (i.e., performance difference between switch and repeat trials). These two indices are thought to reflect different components of cognitive control and have been associated with distinct anatomo-functional brain correlates (e.g. [17–19]). While switching costs reflect local, transient control processes associated with alternation between task sets, mixing costs reflect additional demands on sustained processes involved in maintaining and coordinating multiple task rules. Furthermore, by varying the cue to target interval (CTI), we manipulated task preparation allowing advance preparation during long CTIs (i.e., 1000 ms) but little or no preparation during short CTIs (i.e., 100 ms) [20]. Both switching and mixing cost indices have shown to be sensitive to advance task preparation, in that performance on repeat and switch trials is improved when a cue signaling which task to perform occurs some time before the imperative target (e.g., [18,20]). However, there is not a consensus on the processes that enable such a behavioral benefit afforded by advance task preparation (e.g., task-set reconfiguration, task-set dissipation, cue encoding, etc.; for a detailed review see [21,22]). Moreover, the findings obtained in behavioral, functional magnetic resonance imaging (fMRI) and event-related potential (ERP) studies diverge on the question of whether there are processes underlying task preparation specific to switch trials but not to repeat trials [21,23–25]. In this context, our manipulation of the cue-target interval was not aimed at investigating which of these specific processes will be modulated by the training. Instead, we focused on two types of control adjustments that occur with long and short preparatory intervals: proactive and reactive, respectively [23]. When the cue is presented much earlier than the target, preparation for the next task is possible and a proactive type of control adjustment is more likely to be engaged. Conversely, when the CTI is short, there is not enough time to prepare for the task and its implementation relies more on a post-target reactive type of control [23,26]. Considering that during their training the ATCs had to face the complexity of a radar display and an airport tower simulator, and that they had to learn how to manage this plethora of information, we expected the measures described above to be sensitive to the training-induced modulations. In particular, we predicted we would observe changes in sustained and/or proactive type of control given that, in order to foresee possible aircraft collisions, they had to learn to predict future traffic states and to watch over multiple information simultaneously. Moreover, we expected a more transient and/or reactive type of control to be modulated as well, considering the amount of events they had to manage differently and their high rate of occurrence.

Given the amount of information ATCs have to keep track of, we administered them computerized versions of memory tasks in order to understand whether training-related increases occurred in short-term and/or working memory capacities. Furthermore, resistance to interfering information and conflict resolution were assessed by Stroop tasks that involved selectively attending to one weak feature of the stimulus (i.e., color name), while trying to ignore another prevailing feature (i.e., word meaning). The Stroop effect found in these types of paradigms indicates poorer performance on trials where the two features (i.e., the color and the meaning of a word) are incongruent relative to when they are congruent [27]. This performance cost reflects the ability to maintain activation of task-relevant information in spite of distracting or interfering events. We expected ATCs to be less distracted by task-irrelevant information given that they are trained to cope with a high number of distracting stimuli on the radar screen. For example, despite the fact that different routes are evidenced by different colors within the same sector, they are in charge of paying attention only to some of those routes while ignoring the other ones.

Importantly, whenever a task taxed an explicitly verbal type of process (e.g., color-word Stroop, letter span), an equivalent visuo-spatial version of the task was used to control for possible domain-specific effects due to the nature of the ATC training (i.e., requiring more visuo-spatial abilities [28]), since for some of the measures that we used the two domains were found to be independent (e.g. [29,30]).

It is worth noting that all trainees went through a selection phase that was mainly based on aptitude tests regarding verbal and logical skills, and on group simulation exercises on problem-solving and decision-making. Hence, some differential skills (e.g., in terms of problem-solving, decision-making) could possibly give rise to pre-training advantages. This possibility highlights the importance of a longitudinal approach in which the same experimental group of trainees is tested before and after the training, and is additionally compared to a control group tested with a similar time schedule. To investigate the specific effects of training, the inclusion of a control group is critical in order to characterize changes in the experimental group, above and beyond unspecific learning effects.

Moreover, to avoid possible differences in basic cognitive abilities (i.e., fluid intelligence) between the two groups given the characteristics of the ATC selection phase, we made sure that the control group was matched on a non-verbal, culture-free reasoning test, administered in the pre-training session. Another important source of experimental control is the exposure of the control group with some form of training-like environment, which could ensure that any observed difference in ATCs would be due to the specificity of their training, rather than to its general effects. To that end, we recruited university students from various programs at the beginning of their bachelor or master course.

To summarize, in the present study we examined whether a real-life, control-demanding training, like the one ATCs undergo, can selectively boost some cognitive control strategies and/or memory capacities. Primarily, considering that the focus of their training was on learning to adopt different control strategies and on rapid task implementation, we expected to find enhancement of both proactive and reactive types of cognitive control. Secondly, because of the importance of selecting and manipulating effectively a considerable amount of information in order to create and actualize different strategies of control, we assumed improvements in short-term and working memory, and/or interference resistance and conflict resolution.

## Method

### Ethics Statement

The study was approved by the Ethics Committee of our hospital (Comitato Etico per la Sperimentazione dell'Azienda Ospedaliera di Padova) and was conducted according to the guidelines of the Declaration of Helsinki. On each session all participants gave their written informed consent and were reimbursed 20 euros for their time.

### Participants and Sessions

Twenty-two ATC students from the ENAV Academy, Forlì, Italy (six females, mean age: 25.4 years, SD = 2.28, range: 22–29 years) and 20 students attending various courses at the University of Padova, Italy (six females, mean age: 24 years, SD = 2.48, range: 21–30 years) voluntarily took part in the study. The two groups did not differ significantly regarding age [less than 1.5 years; *t*(40) = 1.85, p = .07]. All but three participants were right-handed (two left-handed from the ATC group, and one ambidextrous from the control group) as assessed by the Edinburgh Handedness Inventory [31], (ATC group average score: 78.6, SD = 30.6, range: -35-100; control group average score: 75.2, SD = 24, range: 0–100), had normal or corrected-to-normal visual acuity and reported normal color vision.

The identical testing session was repeated after an average of 10.9 months (SD = 0.35, range: 10–11) in the ATC group and after an average of 10 months (SD = 0.94, range: 9–12) in the control group. This temporal distance in months, which was significantly different between the two groups [*t*(40) = 3.76, p < .01], can be explained by some organizational constraints, given that the students of the control group belonged to different courses and as such were less available to be retested after a fixed time period, whereas the schedule of the ATC training was very strict limiting the days in which pre- and post-testing sessions could have been performed. In order to control for possible confounding effects, however, the temporal distance between pre- and post-training sessions was used as a covariate in all analyses. Of note, the two groups were also tested in different sites since the testing sessions for the ATCs had to be performed in Forlì, where their course took place, and the control students were tested in Padova, since they were enrolled at the university of that city. However, in both sites the testing sessions were performed in a quiet and naturally illuminated room and the equipment that was used was exactly the same. Importantly, for each group the testing site and the experimental setting were kept constant in the pre- and post-training session.

### Apparatus, Materials and Procedure

In all testing sessions and sites, stimulus presentation and data recording were controlled by the Presentation software (Neurobehavioral Systems, Inc., Albany, CA) for task-switching, color-word Stroop and spatial Stroop tasks, and by E-Prime software [32] for Short-Term Memory (STM) and Working Memory (WM) tasks, running on a Dell Intel Core laptop computer with a 17 inch screen. An intelligence test (see below) was presented in a paper-and-pencil format. All tasks, except from the intelligence test, were administered during the same testing session and the order of presentation was counterbalanced across participants in a Latin-square fashion.

### Intelligence Quotient (IQ) and memory assessment

During the pre-training phase, all participants completed the Italian version of the Raven’s Advanced Progressive Matrices (RAPM) [33], a non-verbal, culture-free test that evaluates abstract reasoning. The test consisted of two sets of items (Set A: 12 items and Set B: 36 items) in which the participants had to complete a visual pattern by choosing among 8 possible missing pieces.

STM and WM, in both verbal and visuo-spatial domains, were assessed during pre- and post-training sessions in order to verify whether the two groups were matched for STM and WM capacities, and to distinguish possible unspecific learning effects from specific training effects on these memory measures.

STM capacity, in both verbal and spatial domains, was assessed by a computerized version of the Letter span and the Matrix span tasks ([34], Italian adaptation [7]). For both tasks the participants had to recall a sequence of items (consonant letters or red squares inside a 4x4 matrix) presented for 1 second each, with a 500 ms of blank screen in between. All memory items were presented centrally on a white background. After all items of a trial were presented, the participants had to insert the items inside a grid reproducing the exact order of their presentation. In the Letter span task the participants made their responses by clicking the cells of a 3 x 4 grid displaying all the possible letters. In the Matrix span task they had to click the cells of an empty 4 x 4 grid. When a mistake was made, they had the possibility to clear the grid by pressing a button, and to re-insert the items. Performance was measured by the sum of all perfectly recalled sequences. For the Letter span task the size of each sequence ranged from 3 to 8 letters, while in the Matrix span task it ranged from 2 to 7 matrices. In both tasks each sequence length was presented randomly 3 times during the whole task, for a total of 18 trials.

In order to assess verbal and spatial WM the automated versions of the Operation span task and the Symmetry span task were used, respectively ([35], Italian adaptation [7]). The basic structure of the tasks was the same as in the STM tasks, except that now prior to each memory item (i.e., letter or red square) participants had to solve a secondary task. In the Operation span task they had to judge whether a math equation was valid, while in the Symmetry span task they had to judge whether a figure was symmetrical or not at the vertical axis. The time limit for the secondary task was determined for each participant based on his/her average response time during the practice session. Once more, at the end of each trial, participants selected the memory items from a grid in the order they were presented. The performance measure was the same as in the STM tasks. The number of errors and time-outs on the secondary task were also recorded. The size of each set of letters ranged from 3 to 7 (total of 15 trials) while the number of matrices in each trial varied from 2 to 5 (total of 12 trials).

### Tasks and Stimuli

#### Color-word Stroop

We administered a shortened version of the color-word Stroop task used by Puccioni & Vallesi [36]. The four Italian color names (“BLU”—blue, “ROSSO”—red, “VERDE”—green, “GIALLO”—yellow) were used as stimuli. These words were colored in one of the four colors: blue, red, green or yellow. Participants were asked to ignore the meaning of the word and attend to its color by pressing with their index and middle fingers of both hands one of four keys on a computer keyboard. The keyboard keys were ‘C’, ‘V’, ‘B’ and ‘N’, and their color-response association was counterbalanced in the following way: respectively, blue, red, green, yellow for half of the participants, and yellow, green, red, blue, for the other half. The color-word association of each stimulus could be congruent (e.g., “ROSSO” presented in red ink) or incongruent (e.g., “BLU” presented in red ink). The color and the meaning of the word in one trial were never repeated on the subsequent trial in either way (color or meaning). This manipulation minimized positive and negative priming confounds (see [36] for details). During the testing session two blocks of 64 trials were administered, with at least 30 congruent trials in each block. On each trial the target word remained in the center of the screen for 500 ms, followed by a 2000 ms blank response screen and an additional inter-trial interval that varied randomly between 250 and 700 ms. All participants completed a training block composed of 16 trials and reached the criterion (10 correct trials) to move onto the experimental blocks.

#### Spatial Stroop

The spatial Stroop task that was used was a shortened version of the one designed by Puccioni & Vallesi [37]. In one of the four quadrants of the screen (upper-left, upper-right, lower-left or lower-right) an arrow appeared. The arrow could point towards one of four directions: upper-left, upper-right, lower-left or lower-right. Participants had to indicate the direction of the arrow while ignoring its position. They had to respond by pressing the following spatially arranged keys of the keyboard: ‘V’ for lower-left, ‘R’ for upper-left, ‘O’ for upper-right and ‘M’ for lower-right. The direction and position of the arrow could be congruent (e.g., lower-right pointing arrow in the lower-right quadrant) or incongruent (e.g., lower-right pointing arrow in the upper-right quadrant). The task and training procedures were same as the ones described in the color-word Stroop task.

#### Task-switching

This paradigm was a modified version of the one used in Rubin and Meiran [18]. At the beginning of each trial a black fixation cross appeared for 1500 ms, followed by the presentation of the cue stimulus (i.e., response–cue interval). Previous studies showed that the influence of passive task-set dissipation on switching costs is minimized when the duration of the response–cue interval is maintained fixed and is ranged between ~1 sec and ~3 sec [38]. There were two types of cues that informed the participant about the upcoming task: the color task cue, which was formed by a row of three colored rectangles (purple, orange and yellow), and the shape task cue, which consisted of a row of three small black shapes (a triangle, a circle and a square). The two possible cue-to-target intervals (CTI), that is, 100 or 1000 ms, were distributed randomly and equally across trials. Below the cue, at the center of the screen, a target stimulus appeared after the CTI. The target could be a heart or a star shape colored in red or blue. Both the cue and the target remained on the screen until a response was detected. Participants were required to respond to either the shape or the color of the stimulus according to the cue by pressing the left or the right arrow key on a computer keyboard with their right index and middle fingers, respectively. The four possible shape-color combinations were associated with the response keys in a counterbalanced order across participants. In case of an erroneous response, a 900 ms long sound stimulus (Windows 7 default “ding.wav” sound) was presented via headphones at a comfortable level for each participant (error-onset delay: 50 ms) providing direct error feedback.

In the first two blocks of trials the participants performed only one task (color or shape) throughout the entire block. The order of the two single-task blocks was counterbalanced across participants and consisted of 6 practice trials and 24 experimental trials each. The final block was a mixed-task block with half of the trials requiring a color judgment and the other half a shape judgment. This block included 10 practice trials followed by 192 experimental trials with a short break at the halfway point. Half of the trials were repeat trials in which the task remained the same as the one performed on the previous trial and half were switch trials in which the task changed with respect to the previous trial.

## Data Analysis and Results

For the task-switching and the two Stroop tasks, accuracy and RT data were treated as follows. Anticipations (RT<120ms; 1 trial) and errors were discarded from response time (RT) analysis. In order to improve normality and reduce skewness, accuracy data and the remaining RT data were arcsine- and log-transformed, respectively. Next, outlier detection was conducted by calculating the average RTs of each individual in each experimental condition and eliminating responses above and below two standard deviations from the corresponding mean. Due to the adoption of this criterion, the following percentages of correct trials were discarded for the ATC and the control group respectively: 4.9% and 5% in the color-word Stroop, 4.3% and 4.7% in the spatial Stroop and 4.1% and 3.8% in the task-switching.

### IQ and Memory tests

In the RAPM, the two groups did not differ in the total number of correct responses in Set A [*t*(40) = 1.66, p = .1] and in Set B [*t*(40) = 0.93, p = .35].

For each of the memory tasks, performance of the two groups was compared before and after the training by means of a 2-way ANCOVA with Group (ATC vs. controls) as a between-subjects factor, Session (pre- vs. post-training) as the within-subject factor and distance between testing sessions as the covariate. No significant effects emerged (all ps>.1), indicating that the two groups were comparable on both STM and WM capacities, and that there were neither learning nor training effects. See Table 1 for the average score of each group and Group × Session interaction p-values.

**Table 1:** Short-term and working memory measures. Average scores (SD) for the two groups in the pre- and post-training session and p-values for Group × Session interactions.

		ATCs		Controls		
Measure		Pre	Post	Pre	Post	Group × Session *p*
STM span	Letter	59.64 (17.71)	60.77 (22.2)	53.25 (12.51)	59.95 (17.92)	0.301
	Matrix	46.5 (11.1)	53.09 (14.66)	42.25 (12.53)	43.15 (13.69)	0.166
WM span	Operation	50.68 (12.4)	51.73 (14.58)	45.6 (13.6)	44.5 (15.51)	0.639
	Symmetry	22.5 (9.7)	24.95 (7.44)	19.5 (7.79)	18.9 (7.18)	0.753

### Color-word & spatial Stroop

Since in the two Stroop tasks the general design was identical, as was also their main purpose (i.e., to measure conflict resolution), analyses were conducted with the Domain as a within-subject factor. A 4-way mixed ANCOVA was conducted for both accuracy and RT data, with Group (ATC vs. controls) as a between-subjects factor, and Session (pre- vs. post-training), Congruency (congruent vs. incongruent) and Domain (verbal vs. spatial), as the within-subject factors and pre-post temporal distance in months as a covariate.

#### Accuracy

**Fig 1:**
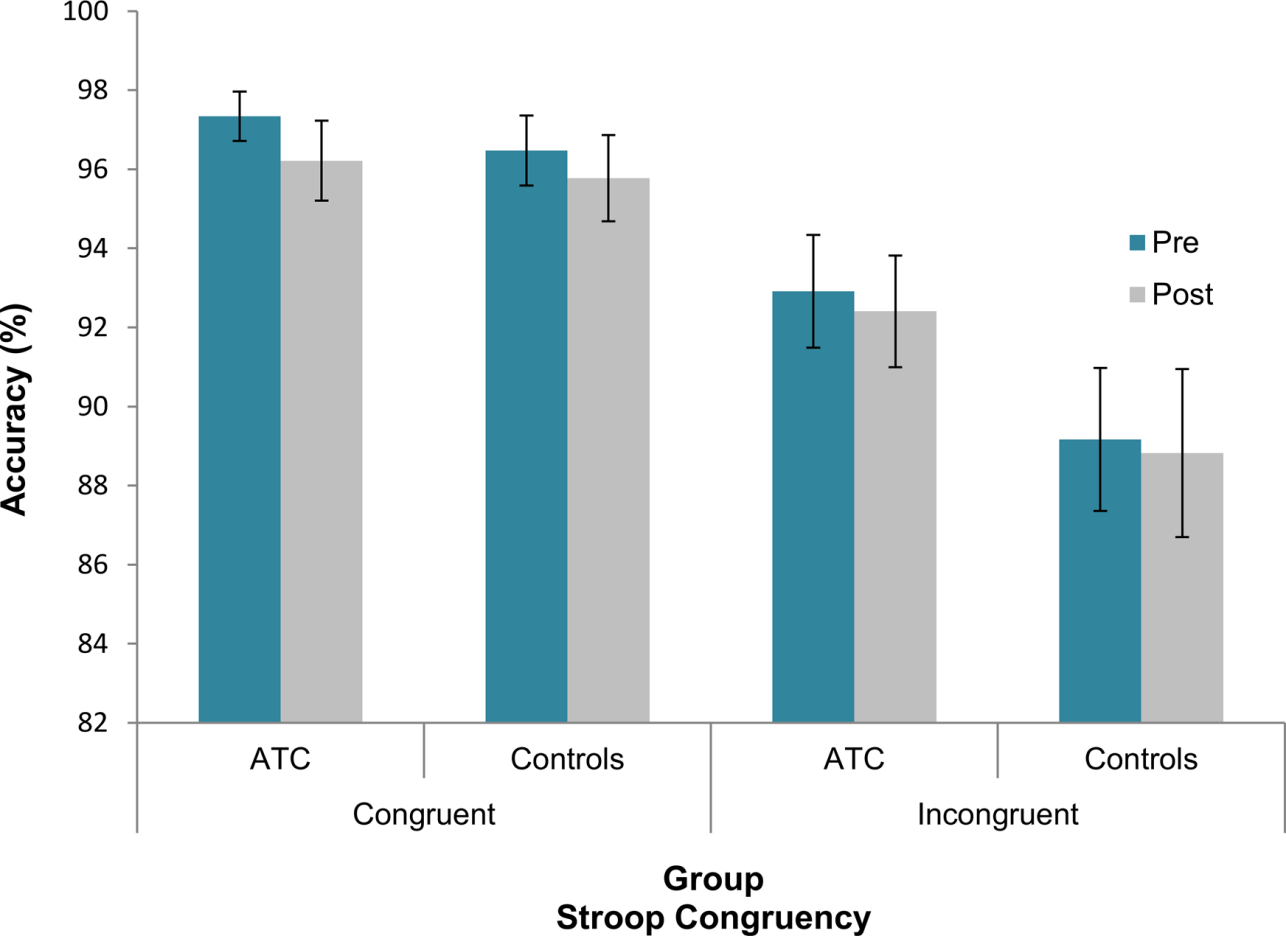
Accuracy scores with standard error (vertical lines) in pre- and post-training sessions across the two groups for congruent and incongruent trials.

The only significant result found, that is, the Congruency × Group interaction [F(1, 39) = 4.5, p = .04, partial *η²* = .1], reflected a smaller difference in accuracy between congruent and incongruent trials (i.e., Stroop interference effect) in the ATC group with respect to the control group (4.11% vs. 7.13%) (see Fig 1; descriptive data are reported in S1 Table).

#### Response Times

Although the main effect of Domain was significant [F(1, 39) = 7.28, p = .01, partial η² = .16] due to longer RTs in the color-word Stroop task when compared to the spatial one (693 ms vs. 484 ms), the Stroop effect was not modulated by the domain (Domain × Congruency interaction p = .26). The significant Domain × Session interaction [F(1, 39) = 4.29, p = .04, partial *η²* = .09] was further modulated by the “distance in months” covariate [F(1, 39) = 4.71, p = .04, partial *η²* = .1]. No other effect was significant. Descriptive RTs data can be found in S2 Table.

### Task-switching

Four analyses were conducted which tested separately single-task trials and repeat trials (i.e., mixing costs) and repeat and switch trials (i.e., switching costs), for both accuracy and RT data. In all four analyses, a 4-way mixed ANCOVA was conducted, with Group (ATC vs. controls) as a between-subjects factor, Session (pre- vs. post-training), CTI (long vs. short), Task condition (single vs. repeat, or repeat vs. switch) as within-subject factors and pre-post temporal distance in months as a covariate. In order to further explore the direction and source of significant interactions, Tukey’s HSD post-hoc tests were used for 2-way interactions, while for more complex interactions only hypothesis-driven pairwise comparisons were performed using Bonferroni adjustment for multiple comparisons.

#### Accuracy

**Mixing Costs:** The analysis showed a significant Session × Group interaction [F(1, 39) = 4.81, p = .03, partial *η²* = .11] which was due to a greater general accuracy improvement in ATCs than in controls. Post-hoc Tukey tests showed an equal performance between the two groups in the pre-training session (Tukey’s p = .7), a better performance only in the ATC group in the post- vs. pre-training session (Tukey’s p < .01) and a trend for a better performance of the ATCs compared to the controls in the post-training session (Tukey’s p = .07).

**Switching Costs:** Similarly to the mixing cost analysis, a significant Session × Group interaction [F(1, 39) = 6.47, p = .01, partial *η²* = .14] was found due to a better general performance in the ATC group. In particular, the accuracy of the two groups was comparable in the pre-training session (Tukey’s p = .99), but the ATC group increased their performance in the post- vs. pre-session (Tukey’s p < .01) becoming significantly more accurate in the post-training session than the controls (Tukey’s p = .02). Moreover, there was a significant CTI × Group interaction [F(1, 39) = 5.34, p = .03, partial *η²* = .12] showing that ATCs had higher accuracy during long CTIs with respect to short CTIs (Tukey’s p < .01), while the controls’ accuracy was not modulated by the CTI (Tukey’s p = .29).

In both accuracy analyses there was no Task × Group interaction (mixing cost: F = .37, p = .54; switching cost: F = .28, p = .59) suggesting that all significant Task × Group interactions found in RT analyses (see below) are not due to trade-offs between speed and accuracy. All descriptive accuracy data are reported in S3 Table.

#### Response Times

**Fig 2:**
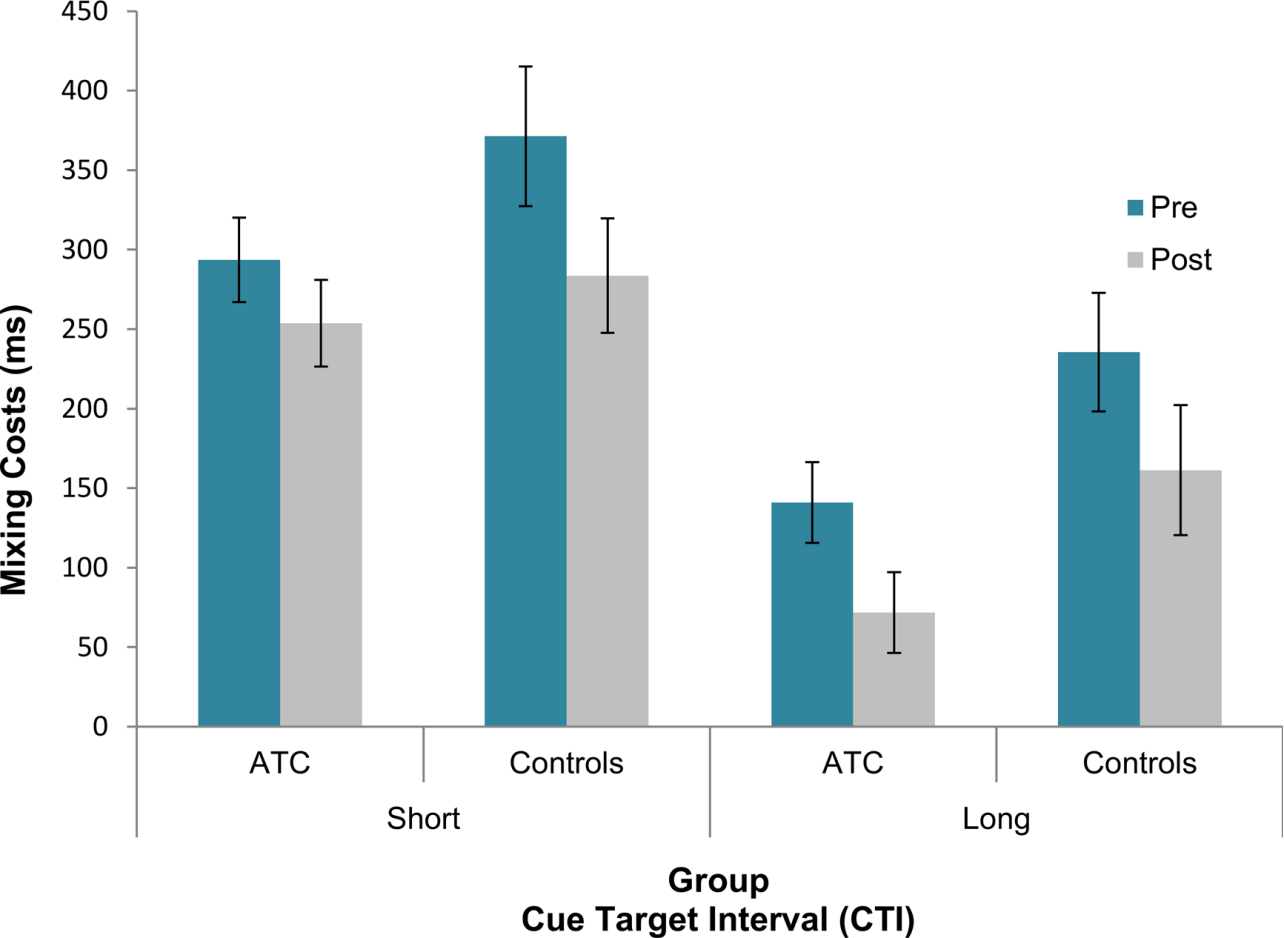
RT mixing costs with standard error (vertical lines) in pre- and post-training sessions across the two groups for short and long CTIs.

**Mixing Costs:** As expected, the main effect of Task [F(1, 39) = 10.18, p < .01, partial *η²* = .21] was significant indicating that RTs were shorter in the single-task trials vs. repeat trials. This effect was also modulated by the “distance in months” covariate [F(1, 39) = 4.86, p = .03, partial *η²* = .11]. The only other significant result was a CTI × Task × Group interaction [F(1, 39) = 5.13, p = .03, partial *η²* = .12]. In order to understand the direction and source of this interaction, mixing costs (i.e., RT difference between single-task and repeat trials) were calculated and contrasted between the two groups for short and long CTIs separately by means of a Bonferroni-corrected pairwise comparison (α/2 < .025). The results showed that mixing costs in the long CTI were smaller in the ATC group, when compared to their controls (ATC: 106 ms vs. controls: 198 ms; p = .017, d = -0.7, CI [-0.23, -0.01]), while mixing costs in the short CTI were virtually the same across the two groups (ATC: 274 ms vs. controls: 327 ms; p = .59, d = -0.18., CI [-0.14, 0.09]) (see Fig 2).

**Fig 3:**
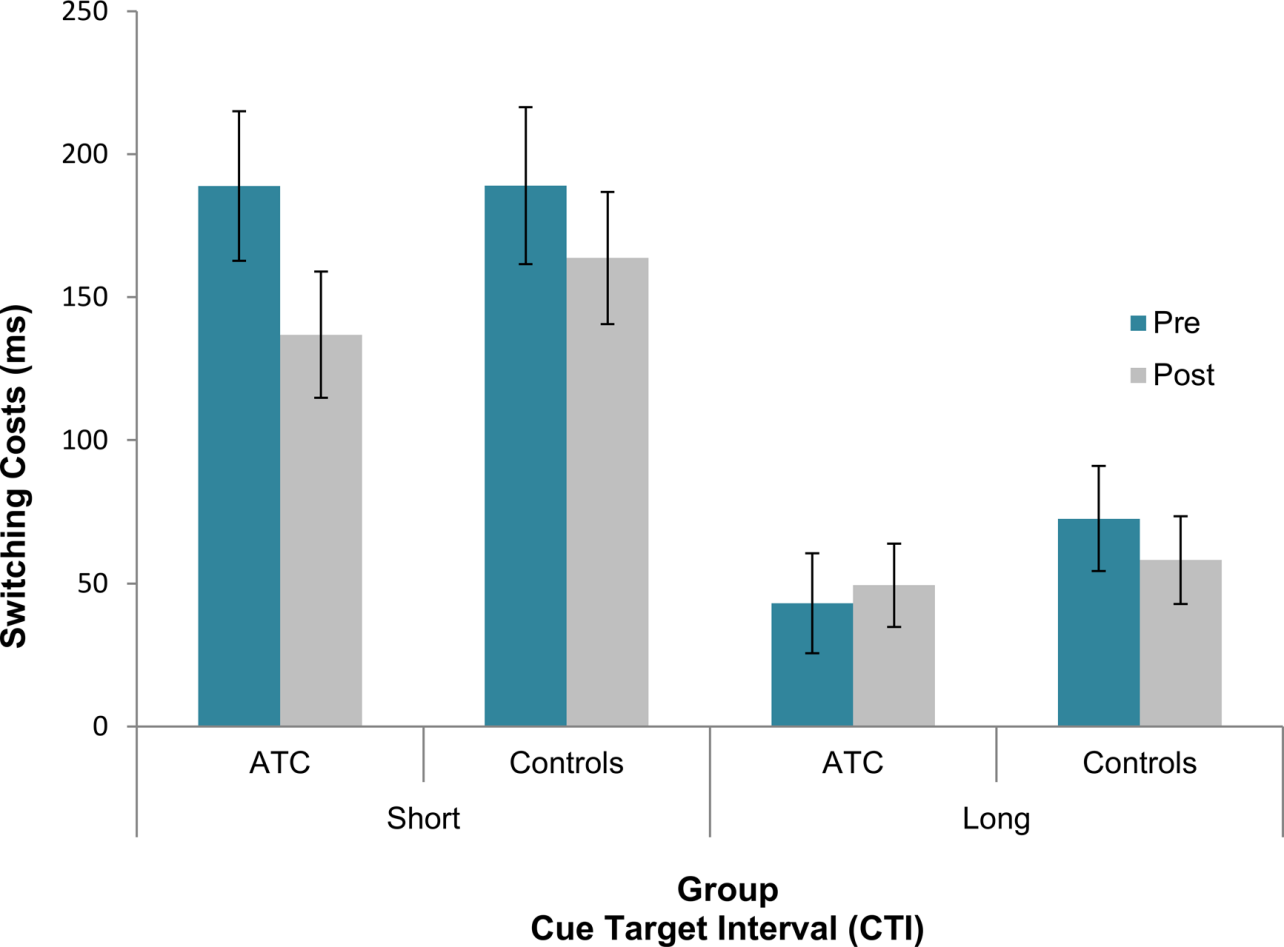
RT switching costs with standard error (vertical lines) in pre- and post-training sessions across the two groups during short and long CTIs.

**Switching Costs:** The analysis conducted on repeat and switch trials revealed a significant interaction between Session, CTI, Task and Group [F(1, 39) = 5.92, p = .02, partial *η²* = .13]. In order to explore this 4-way interaction more closely, the switching cost (i.e., RT difference between switch and repeat trials) training-dependent decrease (i.e., switching cost difference between session 1 and 2) was calculated. This measure was contrasted between short and long CTIs for the two groups separately by means of a Bonferroni-corrected pairwise comparison (α/2 < .025). The results showed that the difference in the training-dependent decrease in switching costs between the short and long CTI was stronger in the ATC group (short CTI pre—post = 51 ms vs. long CTI pre—post = -6 ms, difference = 57 ms; p = .016, d = 0.54, CI [-0.14, -0.01]) than in the control group (short CTI pre—post = 25 ms vs. long CTI pre—post = 14 ms, difference = 11 ms; p = .66, d = -0.1, CI [-0.06, 0.08]; see Fig 3). In other words, this result indicates that, whilst in the ATC group the training-dependent decrease in switching costs was specific for the short CTI, in the control group the same effect did not differ across the two CTI conditions. All descriptive RTs data are reported in S4 Table.

## Discussion

The main objective of this study was to understand whether a real-life, cognitively effortful training, like the one ATCs undergo, has an impact on higher cognitive processes, and, if so, on what specific components. By using an IQ measure, memory tasks, Stroop and task-switching paradigms we aimed at exploring these different measures of cognitive functions in a highly selected and, subsequently, specifically trained group of participants, comparing their performance, before and after the ATC training, to that of a matched group of controls undergoing a more general university training.

The two groups did not differ with each other in terms of general intelligence, which was assessed by the Raven’s intelligence test. No significant difference between the two groups was found for short-term and working memory measures (assessed in the verbal and spatial domains) nor was there any significant change in memory capacities after the training period, even though, at least numerically, the ATCs’ scores were higher both before and after the training (see Table 1). Therefore, we can infer that the results we obtained on Stroop and task-switching tasks cannot be interpreted as due to differences in IQ or to pre-existing advantages and/or changes in short-term and working memory in our samples, despite some previous evidence of far transfer training effects among tasks that tap fluid intelligence, memory and executive functions [39].

In both verbal and spatial versions of the Stroop task, the ATCs’ accuracy, with respect to controls’ one, suffered less from the interference on incongruent trials. This advantage of the ATC group suggests a more efficient coping with the interfering automatized word reading activity. In the literature this Stroop effect reduction has usually been observed during conditions of higher probability for incongruent trials [40,41], and it has been attributed to a proactive control process that prepares the system in advance for the upcoming target [42–44]. Even though in our study the proportion of congruent and incongruent trials was roughly equal, the ATCs probably remained more focused on naming the color irrespective of the type of trial, indicating that the more efficient interference resolution observed in the ATC group depended on a sustained maintenance of task rule across trials. The idea that ATCs are more efficient in maintaining active goal representations prior to their execution is supported also by the results from the task-switching paradigm concerning the mixing costs. During long CTIs, when there was enough time for advance task preparation, the ATCs’ accuracy was higher, and their RTs were less affected by the necessity to manage and coordinate multiple task-sets (i.e., mixing costs). This finding goes along with their better engagement in proactive type of control, since they outperformed the control group only during long CTIs while, during short ones where proactive control could not have fully developed, their performance was comparable.

However, this preparation advantage within interference and switching contexts was present both before and after the training session and, therefore, it probably represents a pre-existent characteristic of the group being selected for the ATC training. As referred to in the Introduction, the selection phase for the training was based on aptitude tests that mainly concerned logical and verbal skills, and on a group simulation exercise where participants had to adopt problem-solving and decision-making abilities that required them to manage multiple task-sets and plan ahead their solving strategies. Although we did not find any advantage in reasoning skills, as measured by the RAPM, some specific skills and traits that were evaluated during the selection phase might have contributed also to these cognitive control differences already present at the baseline.

Unlike proactive control, the training that the ATCs underwent had an impact on more transient and reactive control processes, namely, after the training phase the ATC group showed a specific decrease of switching costs during short CTIs only. As it was discussed previously, while during long CTIs a more proactive type of control is necessary for better task preparation and execution, during short CTIs there is no possibility for this type of control to develop. Accordingly, only reactive control is possible, which is described as that process in charge of stimulus-driven task detection and resolution [1]. The fact that after their training the ATCs decreased the cost of switching to another task selectively during short CTIs purports the idea of training-related gain of reactive control processes. Furthermore, this finding goes in line with switching costs being mainly dependent on reactive capacities or, as already known, on more transient control mechanisms [17].

Another possible interpretation of our results, as pointed out by an anonymous reviewer, is that following their training the ATCs improved the reconfiguration process of the task set [18] and, as such, their improvement was noticeable only on short CTIs, even though it was probably present also on long CTIs. In future work, more specific measures to track task configuration processes (e.g., ERP data) would allow to better explore whether their improvement is specific to short CTIs or partially occurs also on long CTIs. It is interesting to note, however, that in the literature there is some strong evidence that “prepared and unprepared task-switching may, under certain conditions, involve qualitatively distinct mechanisms, rather than simply a temporal shift in a discrete ‘reconfiguration’ process (quote p.227 [45])”. Moreover, according to Ruge and colleagues [23], while during prepared trials the influence of target-related bottom-up information on task-goal activation is weakened, during unprepared trials this bias remains strong. Therefore also the reactive adjustment needed to overcome this bottom-up interference is different between the two types of trials. The fact that we did not observe any change of residual switching costs (i.e., the part of the switching costs that remains during prepared trials) goes in line with possible distinct mechanisms underlying task switching on prepared and unprepared trials. Supporting our claim, other studies have evidenced a dissociation of residual switching costs and full switching costs (see, [46]).

Finally, after the training session, the ATCs’ accuracy improved significantly in both single and task-switching contexts. Considering that this effect was not modulated by the CTI duration nor by the task condition, it could reflect a general change in decisional processes that start only after the arrival of the target (i.e., stimulus categorization and response selection). This result corroborates the earlier suggested idea that the training the ATCs underwent had an impact on the reactive control process (i.e., task implementation) recruited only after target onset. Besides, this accuracy improvement was not followed by a change in RTs, indicating that although they adopted a response strategy that favors accuracy, speed was not sacrificed.

Recently, Yildiz and colleagues [47] conducted an interesting cross-sectional study on airplane pilot trainees that supports the improvements we observed in the ATCs. In particular, the authors observed a superior performance in the pilot group with respect to a control group only in the condition where they had to process two simultaneously triggered actions (i.e., stopping an ongoing response and shifting towards another response). By contrast, when the two actions were temporally separated, the two groups performed equally. The authors attribute this advantage to a more efficient mode of action cascading and stronger attentional processes. Specifically, the different performance in conditions of overlapping stop and change signals is explained in terms of different processing modes, namely serial and parallel modes [48,49]. Notwithstanding all the differences between the experimental designs and populations in this study on pilots and in the present one on ATCs, the distinction of the two processing strategies suggested by Yildiz and colleagues [47] could account for the specific training-related switch cost reduction we observed at short CTIs. Indeed, ATCs processing mode could have improved or changed after the training phase. Future studies combining our longitudinal approach with more sophisticated measures of physiological mechanisms as those used by Yildiz and colleagues [47] should be conducted to elucidate the neurophysiological and neurobiochemical processes that underlie experience-based modulation of cognitive control functions.

Another important issue that the results of this study clarify is the separation of the processes underlying mixing and switching costs and their corresponding association with proactive and reactive type of control, respectively. Developmentally, the processes responsible of mixing and switching costs have been dissociated differently in older adults and children. While mixing costs were found to be more strongly affected by old age than switching costs [50], ADHD in children has been associated with excessively large switching costs but normal mixing costs [51]. In this study, we also added that regardless of the training phase, the ATC group had smaller mixing costs with respect to the control group in the condition when they could prepare for the task at hand, that is, at the long CTIs. On the other hand, switching costs were reduced only after the training phase, and exclusively during unprepared trials (i.e., short CTIs). Thus, for the first time, at the best of our knowledge, a dissociation of the processes underlying mixing and switching costs has been observed longitudinally, as a result of training, within the same group of participants. Moreover, the decrease of mixing and switching costs, that was observed respectively during long and short CTIs, confirms a partial overlap between the processes underlying a proactive type of control with those causing mixing costs, and the processes underlying reactive type of control with those causing switching cost, as suggested by Braver and colleagues [17].

Another important point of this study is that it discriminates training-related changes from a group’s inherent advantage that, if the students had not been tested longitudinally, could have been misleadingly interpreted as an acquired ability. Furthermore, it is important to underline that the observed longitudinal changes were present only in the ATC group and therefore were not effects of practice, as usually found in studies showing improvements in task switching paradigms that use task switching-like trainings [52,53].

In this study we show how real-life training, focused on creating skills necessary for one of the most cognitively effortful jobs, modulates specific cognitive control processes. ATCs’ most relevant skills can be described in three main points: 1) the ability of planning control strategies based on situational conditions, 2) the ability of implementing fast and accurate control strategies, 3) the ability of adapting their strategies to deal with unexpected events [54]. In terms of cognitive control processes, the first ability points out the necessity of developing proactive control strategies that allow controllers to plan ahead the optimal controlling strategy to suit a given situation. In this study, we observed that the students enrolled in the ATC training relied more consistently on proactive control strategies by selecting more efficiently task-relevant information and maintaining task goals. This advantage was not influenced by the training phase and it was probably one of the characteristics looked for during the selection phase. Based on this finding we can speculate that paradigms requiring proactive type of control, like the task-switching with long CTIs and the Stroop task used here, could provide a useful measure during the selection phase of central abilities necessary in ATC. The second and third abilities concerning implementation and adaptation of ATC strategies, on the other hand, probably require recruitment of reactive control, given that both activities are triggered by relevant and/or unexpected sources of bottom-up information and are highly time-constrained. These specific job characteristics fit well with our finding of training-induced improvement of reactive control. By boosting reactive processes, it is likely that controlling becomes less vulnerable to attentional captures that could disrupt event-driven goal activation, a crucial feature of the ATC job.

Possible limitations of our study include a relatively low number of volunteers that applied for the study and complied with it until the second session. Please note, however, that in general students attending the whole ATC course in Forlì were not many more than those successfully tested (i.e., about 40 in total) and that this course is not regularly offered annually nation-wise but on a very occasional basis. Another limit, which we controlled for with the use of ANCOVAs, was the fact that the schedule for testing availability was very strict for the ATCs and caused small differences between the testing periods of the two groups.

Despite these limits, our study showed a dissociation between proactive and reactive control processes in the ATC group that paves the way for future work. For example, to corroborate and extend the present findings, future studies should use other more specific markers of proactive and reactive control processes (e.g., AX version of the Continuous Performance Test) in order to understand how specific job-related abilities, assessed both longitudinally and in highly trained experts, can account for different inter-individual reliance on cognitive control strategies. According to the Dual-Mechanism of Control (DMC) model [1], a tendency to adopt a proactive or reactive control strategy has been found to be useful in understanding intra- and inter-individual variability in cognitive control. Although within the DMC framework changes in cognitive control strategy should be observed as shifts from one type of control to another, the proposed model does not exclude that these control modes are partially independent and could both be boosted selectively. In this study, we observed that an experience of real-life training boosted reactive control processes within a group that already relied more on proactive control strategies before training. This experience-based enhancement of cognitive control shows the extent to which executive functions can be shaped by the type of activity we are immersed in and, most importantly, reveals the importance of life experiences on explaining inter-individual differences in cognitive functioning.

## Supporting Information

S1 Table Verbal and spatial Stroop accuracy (%). Average accuracy scores (SD) for the two groups in pre- and post-training sessions on congruent and incongruent trials.
(DOCX)

S2 Table Verbal and spatial Stroop RTs (ms). Average RTs (SD) for the two groups in pre- and post-training sessions on congruent and incongruent trials.
(DOCX)

S3 Table Task switching accuracy (%). Average accuracy scores (SD) on single-task, repeat and switch trials for the two groups in pre- and post-training sessions, on long and short CTIs.
(DOCX)

S4 Table Task switching RTs (ms). Average RTs (SD) on single-task, repeat and switch trials for the two groups in pre- and post-training sessions, on long and short CTIs.
(DOCX)
