# Supplementary material for: How Life Experience Shapes Cognitive Control Strategies: The Case of Air Traffic Control Training
Source: PLoS One. 2016 Jun 16;11(6):e0157731. doi: 10.1371/journal.pone.0157731 (PMC4911060; doi:10.1371/journal.pone.0157731)
Supplement: S1 Table — Average accuracy scores (SD) for the two groups in pre- and post-training sessions on congruent and incongruent trials. (DOCX) [file pone.0157731.s001.docx]

S1 Table.

|  |  | ATCs | | Controls | |
| --- | --- | --- | --- | --- | --- |
| Measure (Accuracy) | | Pre | Post | Pre | Post |
| Verbal | Congruent | 95.19 (5.18) | 93.22 (8.76) | 93.99 (7.47) | 93.44 (7.37) |
|  | Incongruent | 92.44 (7.92) | 90.72 (8.68) | 88 (8.36) | 87.08 (10.52) |
|  |  |  |  |  |  |
| Spatial | Congruent | 99.49 (0.91) | 99.21 (1.16) | 98.97 (1.95) | 98.11 (3.58) |
|  | Incongruent | 93.4 (6.32) | 94.1 (6.67) | 90.33 (12.15) | 90.56 (9.3) |
