# Supplementary material for: How Life Experience Shapes Cognitive Control Strategies: The Case of Air Traffic Control Training
Source: PLoS One. 2016 Jun 16;11(6):e0157731. doi: 10.1371/journal.pone.0157731 (PMC4911060; doi:10.1371/journal.pone.0157731)
Supplement: S2 Table — Average RTs (SD) for the two groups in pre- and post-training sessions on congruent and incongruent trials. (DOCX) [file pone.0157731.s002.docx]

S2 Table.

|  |  | ATCs | | Controls | |
| --- | --- | --- | --- | --- | --- |
| Measure (RTs) | | Pre | Post | Pre | Post |
| Verbal | Congruent | 627 (143) | 592 (106) | 711 (205) | 623 (144) |
|  | Incongruent | 745 (171) | 681 (142) | 826 (234) | 747 (201) |
|  |  |  |  |  |  |
| Spatial | Congruent | 441 (86) | 409 (51) | 490 (93) | 421 (51) |
|  | Incongruent | 533 (106) | 474 (59) | 598 (147) | 507 (82) |
