# Supplementary material for: How Life Experience Shapes Cognitive Control Strategies: The Case of Air Traffic Control Training
Source: PLoS One. 2016 Jun 16;11(6):e0157731. doi: 10.1371/journal.pone.0157731 (PMC4911060; doi:10.1371/journal.pone.0157731)
Supplement: S3 Table — Average accuracy scores (SD) on single-task, repeat and switch trials for the two groups in pre- and post-training sessions, on long and short CTIs. (DOCX) [file pone.0157731.s003.docx]

S3 Table.

|  |  | ATCs | | Controls | |
| --- | --- | --- | --- | --- | --- |
| Measure (RTs) | | Pre | Post | Pre | Post |
| Short CTI | Single | 95.08 (5.7) | 97.16 (4.53) | 96.88 (4.46) | 96.88 (2.98) |
|  | Repeat | 95.36 (4.21) | 96.97 (3.32) | 94.9 (4.71) | 95.42 (4.99) |
|  | Switch | 90.15 (6.84) | 93.75 (5.49) | 90 (9.4) | 89.06 (11.31) |
|  |  |  |  |  |  |
| Long CTI | Single | 96.78 (3.62) | 99.05 (1.79) | 98.33 (2.09) | 97.5 (2.84) |
|  | Repeat | 95.64 (4.4) | 98.96 (2.11) | 95.63 (6.76) | 95.73 (5.17) |
|  | Switch | 94.89 (6.26) | 97.73 (2.8) | 93.02 (7) | 91.77 (6.88) |
