# Supplementary material for: How Life Experience Shapes Cognitive Control Strategies: The Case of Air Traffic Control Training
Source: PLoS One. 2016 Jun 16;11(6):e0157731. doi: 10.1371/journal.pone.0157731 (PMC4911060; doi:10.1371/journal.pone.0157731)
Supplement: S4 Table — Average RTs (SD) on single-task, repeat and switch trials for the two groups in pre- and post-training sessions, on long and short CTIs. (DOCX) [file pone.0157731.s004.docx]

S4 Table.

|  |  | ATCs | | Controls | |
| --- | --- | --- | --- | --- | --- |
| Measure (RTs) | | Pre | Post | Pre | Post |
| Short CTI | Single | 450 (72) | 469 (80) | 504 (65) | 477 (80) |
|  | Repeat | 743 (147) | 723 (168) | 875 (219) | 761 (201) |
|  | Switch | 932 (157) | 860 (193) | 1064 (264) | 925 (224) |
|  |  |  |  |  |  |
| Long CTI | Single | 418 (61) | 434 (68) | 451 (55) | 435 (63) |
|  | Repeat | 559 (148) | 505 (151) | 686 (204) | 596 (221) |
|  | Switch | 603 (163) | 555 (169) | 759 (232) | 654 (243) |
